# Driver mutation impact in Chinese hepatocellular carcinoma

**DOI:** 10.1371/journal.pone.0355717

**Published:** 2026-08-24

**Authors:** Ruiheng Wu, Houmin Xing, Jeffrey P. Townsend

**Affiliations:** Department of Biostatistics, Yale School of Public Health, New Haven, Connecticut, United States of America; OMICS, PERU

## Abstract

Hepatocellular carcinoma (HCC) remains a leading cause of cancer mortality in China. The Chinese Liver Cancer Atlas (CLCA) recently reported whole-genome sequencing of 494 HCC tumors, nominating 23 coding and 31 non-coding driver candidates based on combined *P* values across multiple algorithms. However, the statistical significance of recurrence alone does not distinguish functionally impactful drivers from neutral passengers. We therefore analyzed cancer effects in the CLCA dataset, quantifying the selective advantage conferred by each mutation. Whole-genome mutation calls from CLCA were analyzed. Gene- and trinucleotide context-specific neutral mutation rates were estimated for all sites. Scaled selection coefficients for every recurrent variant were quantified. Pairwise selective epistasis between genes was quantified and tested. Associations between selected driver mutations and clinical outcomes were examined in the TCGA-LIHC cohort, which included censoring information. We identified canonical drivers *CTNNB1*, *TP53,* and the *TERT*-promoter hotspot as subject to intense positive selection in the CLCA cohort. We also prioritized several lower-prevalence candidate genes and variants with high inferred cancer effect sizes, including *P4HA1*, *STAT3*, and *IL6ST*. These lower-prevalence events should be interpreted as computationally prioritized candidates requiring variant-level functional validation rather than as established novel HCC drivers. Conversely, some genes previously nominated as driver candidates exhibited cancer effects that were indistinguishable from neutrality. These findings show that not all recurrently mutated genes exert equivalent inferred selective effects. Such evolutionary prioritization complements recurrence based driver discovery by ranking somatic variants according to inferred selective effect, while highlighting the need for larger cohorts and functional studies to validate rare high-effect candidates.

## Introduction

Hepatocellular carcinoma (HCC), which most often develops from chronic liver diseases such as hepatitis B and C infections and cirrhosis, is a leading cause of cancer-related mortality globally, with over half of cases occurring in China [1–4]. To address this threat to health, genomic characterization of HCC is crucial, as it reveals the molecular drivers of tumorigenesis and guides the pursuit of targeted therapies [5]. The recent Chinese Liver Cancer Atlas (CLCA) addressed this need by performing whole-genome sequencing (WGS) of 494 untreated HCC tumors collected between 2017 and 2020 at Eastern Hepatobiliary Surgery Hospital and Shanghai Zhongshan Hospital. Using multiple established algorithms to determine statistical significance, the CLCA study nominated 23 coding and 31 non-coding driver candidates [6]. CLCA used reliable “multi-caller” significance frameworks to nominate both coding and noncoding candidates. Significance-based frameworks provide valuable insights into whether or not specific genes play a role in cancer. However, they remain vulnerable to mutational heterogeneity: long or highly mutable genes can achieve low *P* values because of elevated background rates and higher information content within the data, rather than due to substantial contributions to proliferation and survival of the cancer cell lineage. Ranking by *P* values or by cohort-level frequency thresholds systematically under-identifies rare drivers that are highly impactful within a small number of patients [7–9].

Conceptually, a mini-driver model has been proposed to address the possibility that many somatic mutations are neither classical high-impact drivers nor neutral passengers, but instead confer weak tumor-promoting effects that may collectively contribute to tumorigenesis, particularly in tumors with high mutation burden, genomic instability, or strong mutagenic exposure [10]. Our analysis shares with this model the view that tumor evolution is not fully captured by a binary “driver-versus-passenger” classification. Specifically, we estimated site- and context-specific neutral mutation rates and inferred per-variant cancer effect sizes (scaled selection coefficients). This method decouples mutability from selection and enables a more biologically meaningful prioritization across coding and non-coding loci, including rare, high-effect events at both the gene and variant levels [11]. Importantly, cancer effect size estimates provide insight into inferred positive selection during tumor evolution and can help prioritize genes and variants for follow-up studies of biochemical function, clinical relevance, or therapeutic actionability, although these estimates do not by themselves establish the functional mechanism, prognostic significance, or therapeutic actionability of an individual variant. Our findings extend the mini-driver concept, revealing a continuum of selective effects, including canonical high-effect drivers, rare high-effect candidates, and potentially weaker-effect mutations that all collectively contribute to oncogenesis.

To build on the foundational work of the CLCA and enable direct cross-cohort comparison, we quantified mutation rates and cancer effects using the CLCA and the data from The Cancer Genome Atlas Liver Hepatocellular Carcinoma (TCGA-LIHC) project [12]. We then evaluated associations between selected driver mutations and clinical outcomes in TCGA-LIHC, where survival data and censoring information were available. We quantified the selection intensity of recurrent driver mutations and prioritized high-effect candidate genes and variants. This framework enabled prioritization of recurrent HCC variants by selective effect and identified candidates for functional and clinical follow-up.

## Materials and methods

### The study workflow

**Fig 1:**
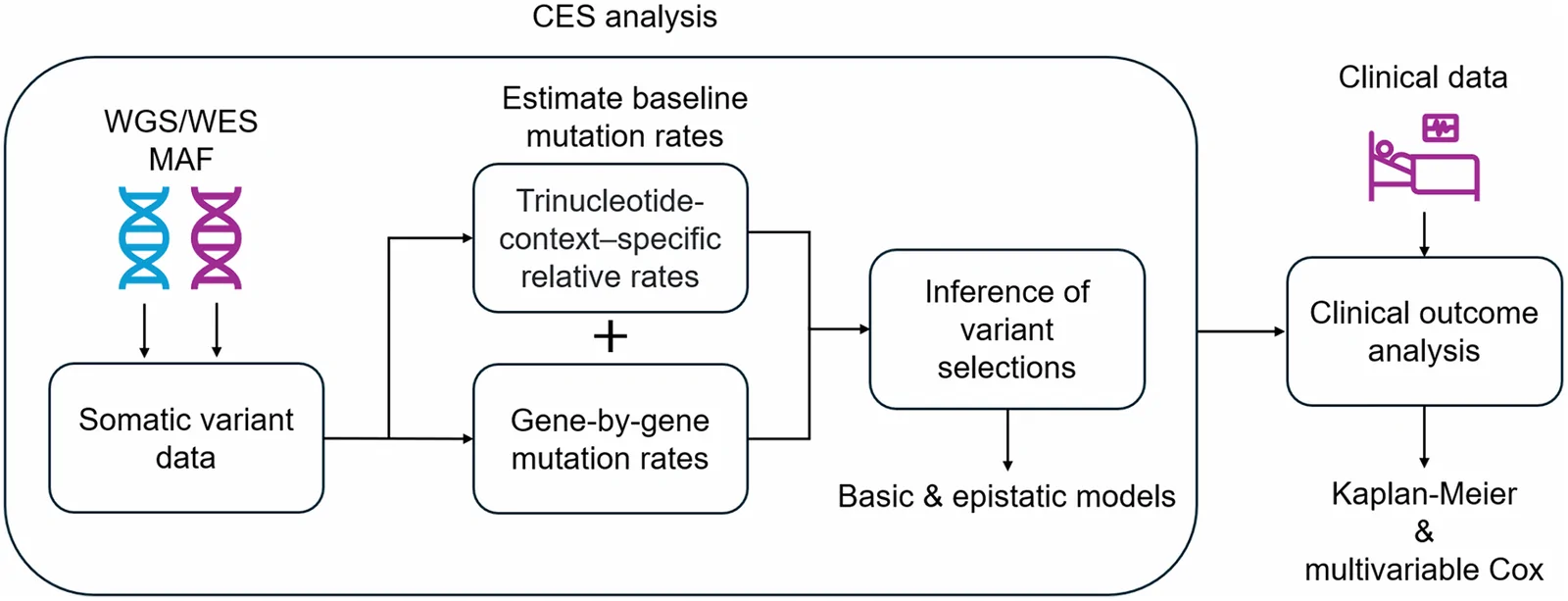
Workflow for estimating sample- and site-specific neutral mutation rates in hepatocellular carcinoma. The workflow identifies potential driver variants, quantifies their effect sizes, and assesses the associated clinical outcomes for each mutation.

Our analysis proceeded in two stages: estimation of cancer effect sizes and evaluation of clinical outcomes (Fig 1). For cancer effect analysis, we executed cancereffectsizeR (v2.10.2) [11]. For clinical outcome analysis, we used the survival package (v3.8-3) to assess associations between mutation status and overall survival [13]. All analyses were performed in R (v4.4.2) using default settings unless otherwise specified.

### Data preparation

We obtained tumor sequence data from the CLCA and from The Cancer Genome Atlas Liver Hepatocellular Carcinoma (TCGA-LIHC) project (**Table 1**) [12]. Somatic variant data were obtained from the raw data_mutations.txt mutation files to be processed to Mutation Annotation Format (MAF) files using *cancereffectsizeR* (v2.10.2) and hg38 reference package ces.refset.hg38. Because the CLCA mutation coordinates were provided relative to hg19/GRCh37, CLCA variants were converted to hg38 using the UCSC hg19ToHg38.over.chain liftOver chain.

**Table 1:** CLCA-HCC and TCGA-LIHC cohorts used for cancer effect size analysis.

Feature	CLCA-HCC	TCGA-LIHC
**Total cohort size**	494 samples; 494 unique patients	379 samples; 377 unique patients
**Analyzable cohort** ^*b*^	493 samples; 493 unique patients; 229,172 filtered MAF records	373 samples; 373 unique patients; 51,310 filtered MAF records
**Sequencing strategy**	Whole-genome sequencing (WGS)^*c*^	Whole-exome sequencing (WXS)^*c*^
**Sequencing depth**	~120×	≥20×
**HBV infection**	94.5%	Not available
**HCV infection**	2.6%	Not available
**Mutation burden**	Median 94 nonsynonymous mutations/sample; median TMB^*a*^ 3.10 mutations/Mb (IQR 2.17–4.87; *n* = 494)	Median 70 nonsynonymous mutations/sample; median TMB^*a*^ 2.33 mutations/Mb (IQR 1.68–3.37; *n* = 370)

^*a*^TMB (tumor mutation burden) was calculated as nonsynonymous mutations per megabase.^*b*^Analyzable sample corresponds to the numbers of samples and mutation records retained after MAF preprocessing and cohort-specific loading into cancereffectsizeR.^*c*^Because CLCA-HCC was analyzed as WGS and TCGA-LIHC as WXS, cross-cohort comparisons were interpreted qualitatively rather than as directly equivalent estimates.

After preloading, records flagged as problematic by *cancereffectsizeR* were removed. To reduce likely germline contamination and mapping artifacts, we excluded variants overlapping common germline-variant sites and removed variants in repetitive regions unless they overlapped a COSMIC Cancer Gene Census site. For TCGA-LIHC, variants located more than 100 bp outside the default exome coverage intervals were also excluded. The resulting filtered CLCA-HCC and TCGA-LIHC MAF files were then used as inputs for cancereffectsizeR [11]. CLCA-HCC was loaded with genome-wide coverage to reflect its origin as WGS data, whereas TCGA-LIHC was loaded with exome coverage to reflect its origin as WXS data, enabling mutation-rate and cancer-effect calculations to account for differences in assayed genomic territory.

Raw somatic variant data and clinical data for these two cohorts were obtained from cBioPortal, deposited in association with the original CLCA-HCC and TCGA-LIHC studies [6,12]. These data were harmonized with the genomic datasets and converted into analysis-ready objects for survival and regression modeling using the *survival* R package.

### Mutation rates under neutrality

Baseline mutation rates are decomposed into the trinucleotide-context-specific relative rates of single-nucleotide variants (SNV) mutations for each tumor sample and cohort-wide gene-specific background rates (Fig 1). To attribute mutations to putative sources, MutationalPatterns [14] was executed on each tumor, supplying COSMIC mutational signatures (v3.4 for hg38) as a reference set [15], excluding signatures annotated as absent, liver artifacts, or treatment-related. The resulting weight vectors defined relative mutation probabilities of all single-base substitutions in their trinucleotide contexts. Signatures annotated as absent, liver artifact, or treatment related were excluded. Gene-specific background mutation rates were estimated using the dNdScv model, which regressed synonymous mutation counts on gene length as well as liver-specific covariates that we supplied, including replication timing, expression level, and chromatin state [16]. Context-specific vectors were multiplied by gene-level rates and distributed across all genomic sites sharing the corresponding trinucleotide, yielding sample- and site-specific neutral expectations (Fig 1) [11].

### Selection inference

Cancer effect sizes were estimated independently for the CLCA-HCC and TCGA-LIHC cohorts. In this framework, the expected number of observed mutations at each site is modeled as a function of the estimated site-specific neutral mutation rate and a scaled selection coefficient, referred to here as the cancer effect size. Selection coefficients were estimated using maximum likelihood by cancereffectsizeR [11]. Confidence intervals were calculated via profile likelihood. This likelihood-based framework distinguishes recurrence attributable to elevated neutral mutability from recurrence attributable to positive selection, allowing rare but strongly selected variants to be differentiated from frequent variants at highly mutable sites [17].

For gene-level epistasis analysis, genes were retained if they had at least three amino-acid-changing variants represented in the variant-level cancer effect output. All pairs of eligible genes were assessed for epistasis using likelihood-ratio tests [18]. Epistasis *P* values were then adjusted using the Benjamini-Hochberg false discovery rate procedure.

### Survival analysis

Clinical relevance of top candidate mutations was evaluated using TCGA-LIHC clinical data alone, because CLCA-HCC lacked censoring information required for survival analysis. Overall survival was defined according to the TCGA-LIHC clinical data dictionary as the time from initial diagnosis to death from any cause. Disease-free survival was defined as the time from initial surgical resection to the first documented tumor recurrence or disease progression. Patients without an event by the last follow-up were treated as censored, consistent with Surv(time, event) coding.

Kaplan-Meier survival curves were constructed for univariate visualization, and survival distributions were compared using log-rank tests. To account for clinical confounding, multivariable Cox proportional hazards models were fitted when covariate data were available, including mutation status and available clinical covariates such as age, stage, ethnicity, and race. Hazard ratios, 95% confidence intervals, and *P* values were reported [13]. Genes were included in survival analysis when mutation-positive and mutation-negative groups were both present with sufficient survival annotation for model fitting.

## Results

### Cancer effect size and epistasis analysis

**Fig 2:**
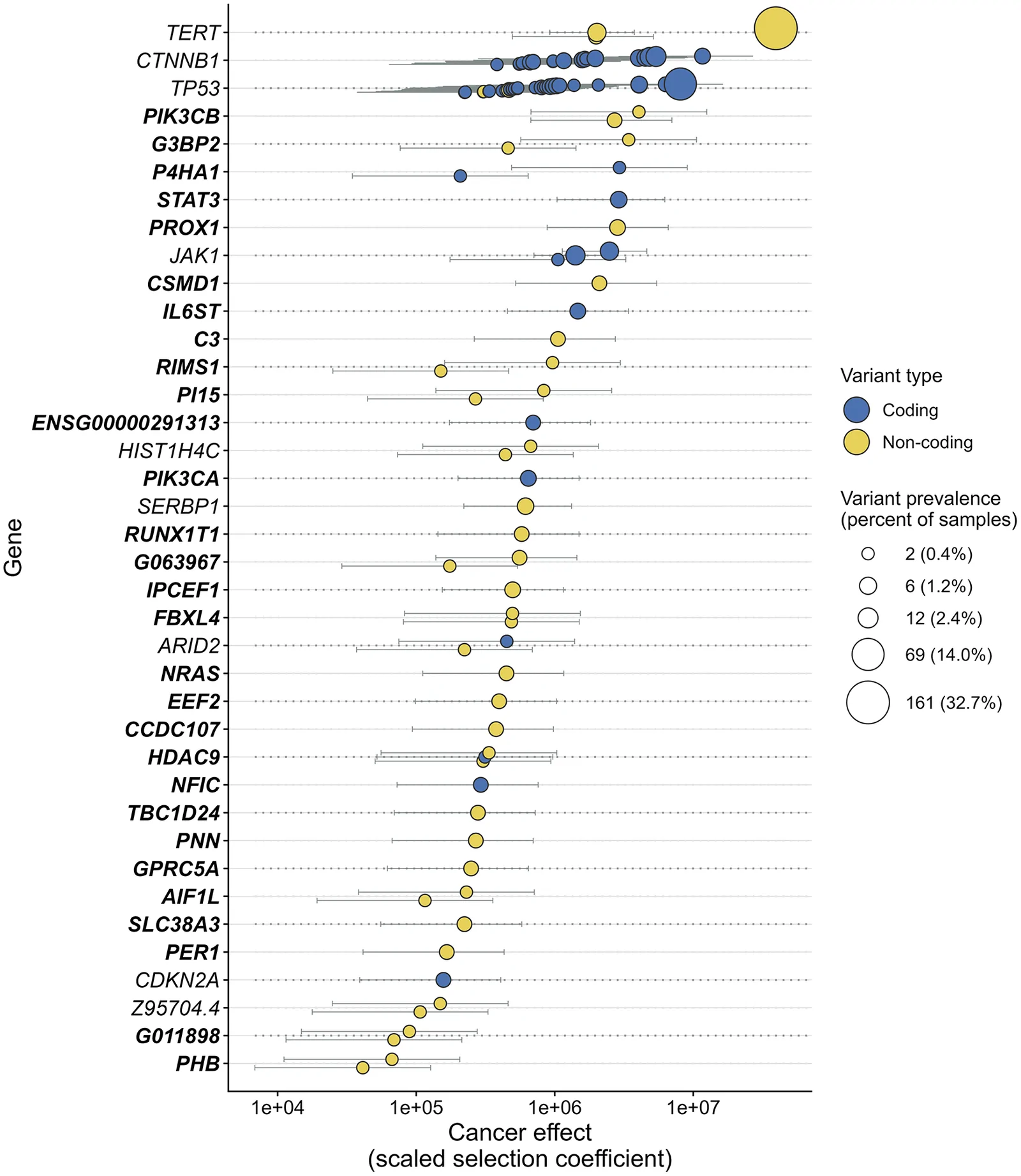
Cancer effect sizes and 95% CIs of recurrent variants in the HCC-CLCA cohort. Variants (circles with diameter proportional to prevalence with fill indicating the variant is in a non-coding (yellow) or coding (blue) region) grouped by gene (≥3 per gene identified in the original CLCA study candidate gene list (plain text), and identified herein (boldface).

Analysis of the HCC-CLCA cohort revealed multiple genes harboring mutations that had been subjected to strong positive somatic selection (Fig 2). As expected, well-established HCC drivers including *TERT*, *CTNNB1*, *TP53*, and *JAK1* exhibited among the highest cancer effects, consistent with previous reports identifying these genes as prominent drivers in both virus-infected and cirrhosis-based HCC [6,19–21]. By decoupling mutation prevalence from underlying cellular mutation rate, this analysis also prioritized high-effect candidate variants in genes that were not emphasized by the original CLCA driver-candidate list. Coding variants in *P4HA1*, *STAT3*, *IL6ST*, *PIK3CA*, and the short, uncharacterized locus ENSG00000291313 exhibited significant inferred positive selection. ENSG00000291313 maps to chromosome 14q32.32 near the q-arm telomeric end, overlapping the broader EIF5 genomic neighborhood on the GRCh38/hg38 reference genome. No clear expression or disease-associated functional annotation is currently available. Among non-coding variants for a well-known gene associated with longevity, *TERT* promoter mutations were predominant, exhibiting both the highest prevalence (more than 32.7% of tumors) and the largest effect sizes. Beyond *TERT*, measurable positive selection was also observed in non-coding regions linked to *PIK3CB*, *G3BP2*, and *PROX1.*

**Fig 3:**
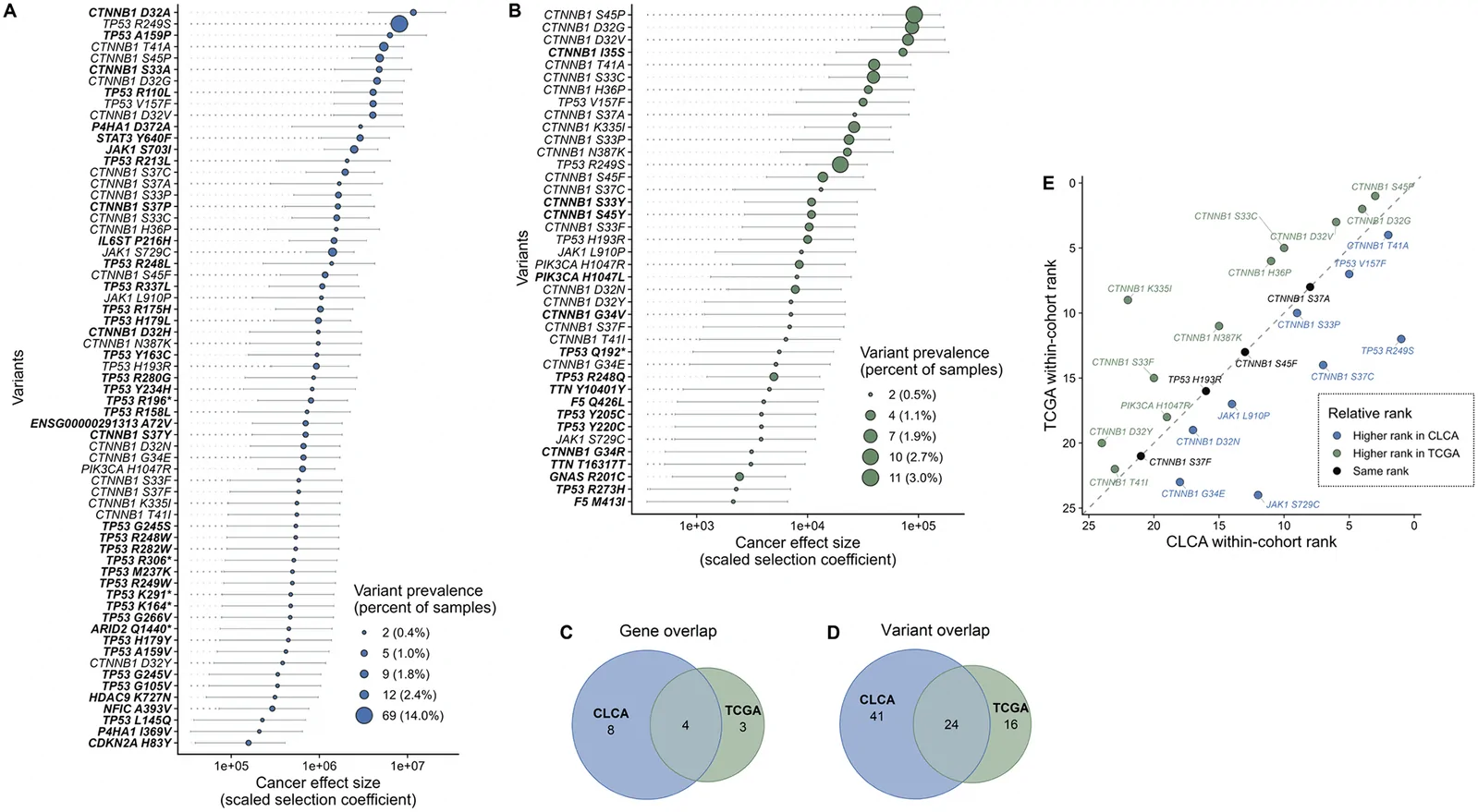
Cross-cohort comparison of amino-acid–changing driver-effect estimates in CLCA-HCC and TCGA-LIHC. Cancer effect sizes of recurrent amino-acid–changing variants were estimated separately in the (**A**) CLCA-HCC and (**B**) TCGA-LIHC cohorts. Variants are ranked by estimated cancer effect size within each cohort. Points indicate maximum-likelihood cancer effect estimates, horizontal lines indicate 95% confidence intervals, and point size is proportional to variant prevalence in the cohort. Boldface variant labels indicate cohort-specific variants among the displayed high-effect substitutions. The *x* axis is shown on a log_10_ scale. (**C**) Gene-level overlap among genes represented by the amino-acid–changing variants shown in panels A and B. (**D**) Variant-level overlap of amino-acid–changing substitutions between CLCA-HCC and TCGA-LIHC. (**E**) Within-cohort rank comparison for shared amino-acid–changing variants. Lower numerical ranks indicate higher estimated cancer effect within a cohort; variants closer to the dashed diagonal have more similar ranks across cohorts. Blue points indicate variants ranked higher in CLCA-HCC, green points indicate variants ranked higher in TCGA-LIHC, and black points indicate variants with the same rank in both cohorts.

To qualitatively compare driver-effect patterns across cohorts, we compared cancer effect sizes from the CLCA-HCC dataset (Fig 3A) with those obtained from the TCGA-LIHC cohort (Fig 3B), restricting the analysis to amino-acid-changing (AAC) substitutions to reduce biases introduced by differences between WGS and WXS sequencing strategies. In both datasets, TP53 R249S emerged as a top variant with an exceptionally high cancer effect size. Likewise, multiple “hotspot” mutations in *CTNNB1* at codons T41, S45, S33, and S37 ranked among the strongest drivers in both analyses. In our analysis, cancer effect sizes were estimated independently within each cohort; comparisons were interpreted as a relative ranking and overlap analysis rather than as a direct numerical comparison of absolute effect-size magnitudes.

In the HCC-CLCA cohort, the highest-effect AAC variants were dominated by these canonical hotspots in *CTNNB1* and *TP53*. Among them, *TP53* R249S exhibited both the largest estimated effect and the highest prevalence (~14% of tumors in our series). Additional high-effect AAC variants include *JAK1* S703I, *P4HA1* D372A, and *STAT3* Y640F. In TCGA-LIHC, the highest-effect variants were also dominated by *CTNNB1* and *TP53*hotspots, together with additional recurrent variants in *JAK1*, *PIK3CA*, *GNAS*, *F5*, and *TTN* (Fig 3B). Compared with CLCA-HCC, the displayed TCGA-LIHC variants had lower maximum prevalence, with the largest point corresponding to approximately 3.0% of tumors. At the gene level, 4 genes were shared between the displayed CLCA-HCC and TCGA-LIHC AAC effect sets (*CTNNB1*, *TP53*, *JAK1*, and *PIK3CA*), whereas 8 candidates were specific to CLCA-HCC (*P4HA1*, *STAT3*, *IL6ST*, *ARID2, HDAC9, NFIC CDKN2A*, and uncharacterized ENSG00000291313) and 3 were specific to TCGA-LIHC (*GNAS*, *F5*, and *TTN*) (Fig 3C). At the variant level, 24 AAC substitutions were shared between cohorts, whereas 41 variants were specific to CLCA-HCC and 16 were specific to TCGA-LIHC among the displayed high-effect variants (Fig 3D). The major driver-effect signals observed across independent HCC datasets included multiple shared variants in *CTNNB1* and *TP53.* Rank comparison of the shared amino-acid–changing variants showed partial concordance between cohorts (Fig 3E). Several *CTNNB1* variants ranked highly in both CLCA-HCC and TCGA-LIHC, consistent with possible strong selection on Wnt/β-catenin pathway activation. However, individual variants differed in relative rank between cohorts. Some variants, including *CTNNB1* S45P, D32G, D32V, S33C, and H36P, ranked relatively higher in TCGA-LIHC, whereas variants R249S and V157F in *TP53*; T41A, D32N, G34E and S37C in *CTNNB1*; and L910P and S729C in *JAK1* ranked relatively higher in CLCA-HCC. A smaller set of shared variants, including *CTNNB1* S37A, S45F, and S37F, and *TP53* H193R, showed similar within-cohort ranks. Overall, these results indicate that CLCA-HCC and TCGA-LIHC share a core set of high effect AAC driver variants, especially in *CTNNB1* and *TP53*, while also showing cohort-specific high effect events that may reflect differences in etiologic background, population composition, sequencing strategy, sample size, and statistical power.

To better characterize potential non-additive selective effects, we quantified pairwise selective epistasis among recurrently mutated genes. In the CLCA-HCC cohort, several gene pairs showed nominal evidence of non-independent selection based on likelihood-ratio tests, including interactions involving *CTNNB1*, *TP53*, *JAK1*, *CDKN2A*, *PIK3CA*, *P4HA1*, and ENSG00000291313 (**S1 Fig**). Among the candidates, the inferred antagonistic relationship between *CTNNB1* and *TP53* is especially biologically plausible as it aligns with previously described alternative routes of hepatocarcinogenesis, in which *TP53* alterations are enriched in HBV-associated disease, whereas *CTNNB1* activation is more common in Wnt/β-catenin–driven tumors. However, this signal did not remain significant after multiple-testing adjustment, it should be viewed as a candidate relationship requiring validation in larger cohorts [22]. Indeed, none of these candidate epistatic relationships remained significant after Benjamini-Hochberg false discovery rate adjustment. Therefore, these results should be interpreted as exploratory hypothesis-generating signals rather than definitive epistatic interactions. Statistical significance of epistatic effects of candidate epistatic CLCA pairs was not reproduced in TCGA-LIHC (**S2 Fig**), consistent with limited power, sparse co-mutation counts, and cohort-specific genetic backgrounds.

### Clinical outcome analysis

To explore whether high-effect driver mutations were associated with patient outcomes, we performed survival analyses using TCGA-LIHC clinical data, because survival censoring information was not available for the CLCA-HCC cohort. Genes were selected for survival analysis based on their relevance to the cancer effect size results and their interpretability in the TCGA-LIHC cohort. Specifically, *TP53* and *CTNNB1* were included because they are canonical HCC drivers repeatedly identified in prior HCC genomic studies and ranked among the highest-effect genes in our cancer effect size analyses [6,19,22–24]. *JAK1* was included because it was identified as a high-effect driver in CLCA-HCC and has known biological relevance to *JAK-STAT* signaling in HCC [6,20]. We also included *P4HA1*, *STAT3*, and *IL6ST* because they emerged as lower prevalence candidate high effect genes in the CLCA-HCC cancer effect analysis and represent biologically plausible pathways related to extracellular-matrix remodeling, inflammatory signaling, and IL-6/gp130/JAK/STAT pathway activation [21,25–27]. These genes were therefore chosen to evaluate whether inferred cancer-cell selective advantage corresponded to detectable clinical outcome differences in an independent cohort. For each selected gene, patients were classified as mutation-positive if their tumor carried at least one nonsynonymous mutation in that gene and mutation-negative otherwise. Overall survival and disease-free survival were analyzed separately. Kaplan-Meier curves and log-rank tests were first used to compare survival between mutation-positive and mutation-negative groups. To account for potential clinical confounding, Cox proportional hazards models were then constructed for each gene and endpoint. In these models, gene mutation status was the primary variable of interest, and available clinical covariates were incorporated as adjustment variables, including age at diagnosis, sex, pathologic stage, race, and ethnicity. These covariates were included because demographic background and tumor stage can influence survival outcomes in HCC and may confound the relationship between mutation status and patient prognosis.

Among the selected driver genes tested, *TP53* mutation was associated with worse overall survival in TCGA-LIHC. Patients with *TP53* mutated tumors showed shorter overall survival compared with patients with *TP53* wild-type tumors by Kaplan-Meier analysis, and *TP53* mutation was associated with increased mortality risk in Cox proportional hazards modeling (HR = 1.71, 95% CI: 1.10–2.66, Cox *P* = 0.018; **Table 2**, **S3 Fig** **A**). This association did not remain significant after Benjamini-Hochberg adjustment across the tested genes (BH-adjusted *P* = 0.088). In contrast, *TP53* mutation was not significantly associated with disease-free survival in the Cox model (HR = 1.31, 95% CI: 0.88–1.97, Cox *P* = 0.184; **Table 2**, **S3 Fig** **B**). Similarly, mutations in *CTNNB1*, *JAK1*, *STAT3*, and *IL6ST* did not show statistically significant associations with overall survival or disease-free survival after Cox modeling and multiple-testing adjustment (**Table 2**). Although *IL6ST* showed a nominal association with overall survival in the univariate log-rank analysis, this association was not significant in the Cox model or after multiple-testing adjustment (**Table 2**, **S3Fig** **G**). For rare candidate genes, survival analysis was limited by the small number of mutation-positive patients. For example, *P4HA1* was mutated in only one TCGA-LIHC patient with usable survival annotation, preventing reliable Kaplan-Meier or Cox model estimation. Therefore, the absence of a significant survival association for rare high-effect candidates should not be interpreted as evidence that these mutations are biologically irrelevant.

**Table 2:** Survival analysis^*a*^ summary for selected driver mutations in TCGA-LIHC.

Gene	Endpoint^*b*^	*n* used^*c*^	*n* used in Cox^*d*^	*n* mutated^*e*^	*n* wild-type^*f*^	Events mutated^*g*^	Events wild-type^*h*^	Log-rank *P* value^*i*^	Cox HR (95% CI)^*j*^	Cox *P* value^*k*^	Cox BH *P* value^*l*^	PH test *P* value^*m*^
*TP53*	OS^*n*^	371	326	106	265	41	91	0.022	1.71 (1.10–2.66)	0.018	0.088	0.329
*TP53*	DFS^*o*^	317	282	90	227	42	103	0.235	1.31 (0.88–1.97)	0.184	0.460	0.235
*CTNNB1*	OS	371	326	93	278	30	102	0.744	1.07 (0.68–1.69)	0.763	0.942	0.787
*CTNNB1*	DFS	317	282	77	240	39	106	0.549	1.07 (0.72–1.59)	0.726	0.824	0.575
*JAK1*	OS	371	326	8	363	2	130	0.808	1.05 (0.25–4.38)	0.942	0.942	0.890
*JAK1*	DFS	317	282	7	310	1	144	0.124	0.25 (0.03–1.80)	0.169	0.460	0.243
*P4HA1*	OS	371	NA	1	370	0	132	NA	NA	NA	NA	NA
*P4HA1*	DFS	317	NA	1	316	0	145	NA	NA	NA	NA	NA
*STAT3*	OS	371	326	5	366	2	130	0.605	2.10 (0.50–8.78)	0.311	0.519	0.665
*STAT3*	DFS	317	282	5	312	2	143	0.846	1.17 (0.28–4.84)	0.824	0.824	0.488
*IL6ST*	OS	371	326	11	360	2	130	0.039	0.30 (0.07–1.25)	0.098	0.245	0.879
*IL6ST*	DFS	317	282	8	309	4	141	0.586	1.22 (0.44–3.35)	0.701	0.824	0.040

*^a^* Survival analyses were performed in TCGA-LIHC using gene-level nonsynonymous mutation status.

*^b^* Endpoint indicates whether the analysis used overall survival (OS) or disease-free survival (DFS).

*^c^* *n* used is the number of patients with usable survival time and event-status information for that endpoint.

*^d^* *n* used in Cox is the number of patients included in the Cox proportional hazards model after endpoint eligibility and model-specific missingness filtering.

*^e^* *n* mutated is the number of analyzed patients whose tumor carried at least one nonsynonymous mutation in the indicated gene.

*^f^* *n* wild-type is the number of analyzed patients whose tumor did not carry a nonsynonymous mutation in the indicated gene.

*^g^* Events mutated is the number of mutation-positive patients who experienced the endpoint event.

*^h^* Events wild-type is the number of mutation-negative patients who experienced the endpoint event.

*^i^* Log-rank *P* values are nominal univariate comparisons accompanying Kaplan–Meier visualization.

*^j^* Cox HR (95% CI) reports the hazard ratio and 95% confidence interval for mutation-positive versus mutation-negative tumors.

*^k^* Cox *P* values were obtained from Cox proportional hazards models.

*^l^* Cox BH *P* values were adjusted using the Benjamini-Hochberg procedure across the tested genes within each endpoint.

*^m^* PH test *P* value reports the proportional-hazards assumption test for the mutation status term.

*^n^* OS, overall survival. For OS, the endpoint event was death.

*^o^* DFS, disease-free survival. For DFS, the endpoint event was recurrence or progression.

NA indicates that the model or statistic was not estimable.

Overall, these exploratory survival analyses support the known adverse prognostic association of *TP53* mutation in HCC, while highlighting the limited statistical power of TCGA-LIHC to evaluate rare candidate drivers such as *P4HA1*, *STAT3*, and *IL6ST*.

## Discussion

Here we quantified the selective impact of somatic mutations in hepatocellular carcinoma, distinguishing potent driver mutations from mutations with substantially weaker inferred effects. This approach complements significance-based driver discovery by estimating the strength of positive selection after accounting for site- and context-specific neutral mutation rates [11]. Our findings support the hypothesis that driver mutations do not contribute equally to oncogenesis, revealing marked heterogeneity in selection strength across both coding and non-coding regions. Absolute effect sizes and several top-ranked variants differed between cohorts. In TCGA-LIHC, peak variant prevalences were smaller than in CLCA-HCC (≤~3% vs ~ 14% in CLCA), and estimated cancer effects were generally 1–2 orders of magnitude lower. Nevertheless, several canonical high-effect variants, particularly in *TP53, CTNNB1* and *TERT,* showed qualitative concordance across CLCA-HCC and TCGA-LIHC. This attenuation may reflect differences in cohort composition, etiologic exposures, mutation burden, sequencing depth, and the more restricted genomic territory assayed by whole-exome sequencing relative to the deep whole-genome design of CLCA [6,12]. Therefore, lower-prevalence events in *P4HA1*, *STAT3*, *IL6ST*, and ENSG00000291313 should be interpreted as cohort-prioritized candidates rather than cross-cohort validated HCC drivers.

Our findings also resonate with the mini-driver model of polygenic cancer evolution. This model proposes that some somatic mutations are neither classical high-impact drivers nor fully neutral passengers, but instead may confer weak tumor-promoting effects that act cumulatively, particularly in tumors shaped by genomic instability, high mutation burden, or strong mutagenic exposure [10]. The original mini-driver framework emphasized that multiple weak-effect mutations may collectively substitute for or modify the effect of major-driver events, especially under mutagenic conditions. Related pan-cancer analyses have also challenged a strict driver/passenger dichotomy by showing that putative passenger mutations can span a continuum of molecular functional impact and may collectively influence tumor phenotypes [28]. In this context, cancer effect size analysis provides a complementary quantitative framework. Rather than assigning mutations to discrete categories such as major drivers, mini-drivers, or passengers, it estimates a continuous distribution of inferred selective effects after accounting for neutral mutation rates. Lower effect recurrent mutations identified in our analysis may not be definitive mini-drivers, but they are consistent with the broader concept that HCC driver evolution involves a spectrum of selective effects.

Our analysis showed strong selection on established HCC drivers such as *TP53*, *CTNNB1*, and *JAK1*, while also identifying high-effect variants in *P4HA1*, *STAT3*, *IL6ST*, and the uncharacterized locus ENSG00000291313 with lower prevalence [6,19–24]. Among these, *STAT3* and *IL6ST*, together with *JAK1* identified in the CLCA report, support the central role of inflammatory and cytokine-driven signaling in HCC evolution and align with prior evidence implicating aberrant IL-6/gp130 -JAK/STAT activation as a major oncogenic program in hepatocytes [21,25,29,30]. However, the degree of variant-specific support differs across this pathway. JAK1 S703I has the strongest variant-level support among this group. Prior HCC work showed that *JAK1* S703I activates JAK–STAT signaling, promotes cytokine-independent proliferation, and is associated with sensitivity to JAK1/2 inhibition in a *JAK1* S703I mutant HCC patient derived xenograft model [20]. Therefore, *JAK1* S703I can be described as a previously reported activating HCC-associated mutation.

By contrast, STAT3 and IL6ST require more nuanced interpretation than the canonical HCC Drivers. Extensive literature on HCC supports the importance of IL-6/JAK/STAT3 signaling in hepatocarcinogenesis, tumor progression, and metastatic behavior [21,25,29,30]. Consistent with this biology, a recent East Asian HCC genomic analysis reported *JAK1* S703I, *JAK1* S729C, and *STAT3* Y640F as recurrent alterations with possible population-specific enrichment, with a comment emphasizing the need for validation in larger cohorts [31]. *STAT3* Y640F has plausibility as a driver because activating *STAT3* mutations have been described in inflammatory hepatocellular adenomas, but direct functional evidence for *STAT3* Y640F remains limited [32]. In comparison, the *IL6ST* variant prioritized in our analysis, P216H, has stronger variant-level support. *IL6ST* encodes gp130, the common signal-transducing receptor subunit for IL-6-family cytokines, and activating *IL6ST* alterations are well established in inflammatory hepatocellular tumors [33]. *IL6ST* P216H has been identified previously in inflammatory hepatocellular adenoma and was included in functional studies demonstrating constitutive, IL-6-independent activation of downstream JAK–STAT signaling by tumor-derived gp130 mutants [34]. The high inferred cancer effect of *IL6ST* P216H in the CLCA-HCC cohort has both variant-specific and pathway-level biological support. However, the strongest published evidence for *IL6ST* P216H derives from inflammatory hepatocellular adenoma rather than conventional HBV-associated HCC, and its biological and clinical consequences in HCC therefore remain uncertain. Accordingly**,** our results support *STAT3* Y640F and *IL6ST* P216H as biologically plausible candidate driver variants whose high inferred cancer effects warrant further investigation aiming to test for direct functional roles in HCC.

*P4HA1* is also a biologically plausible candidate high-effect gene. *P4HA1* encodes the alpha subunit of prolyl 4-hydroxylase, an enzyme involved in collagen maturation and extracellular-matrix remodeling. Prior studies have implicated *P4HA1* expression in tumor growth, invasion, extracellular-matrix biology, and hypoxia-associated cancer phenotypes [26,27]. However, to our knowledge, the specific *P4HA1* D372A variant identified in our analysis has not been experimentally validated as a recurrent functional HCC mutation. The high inferred cancer effect of this variant should be interpreted as evidence of positive selection warranting further mechanistic investigation.

The finding that the short, uncharacterized coding gene ENSG00000291313 exhibited a high cancer effect requires cautious interpretation. This locus lies near the *EIF5* genomic neighborhood, with nearby H3K27ac and ENCODE candidate cis regulatory annotations in the GRCh38/hg38 reference, raising the possibility that mutations assigned to ENSG00000291313 may reflect selection on a local regulatory element rather than on the annotated transcript itself. Therefore, the biological interpretation of the observed high cancer effect and especially its attribution to the coding sequence ENSG00000291313 remains provisional.

Tumor suppressor gene *CDKN2A* was identified as an important driver in our analysis as well as in the CLCA and many other studies. Its mutation has been recognized to deregulate cell-cycle progression in HCC [6,35,36]. *PIK3CA*, which encodes the p110α catalytic subunit of PI3K, also showed evidence of positive selection. Activation of the PI3K/AKT/mTOR signaling pathway through *PIK3CA* mutation can promote cell growth, survival, metabolism, and migration [37], and has been implicated in HCC oncogenesis [38]. *CDKN2A* and *PIK3CA* mutations are biologically plausible contributors to HCC evolution that affect complementary processes: cell-cycle control and pro-survival proliferative signaling.

Pairwise epistasis analyses suggested potential interaction patterns involving *CTNNB1*–*JAK1*, ENSG00000291313–*P4HA1*, and *CTNNB1–TP53* (**S1 Fig**)*.* Mutual exclusivity between *CTNNB1* and *TP53* mutations has led to an expectation of reciprocal antagonistic epistasis, in which the selective advantage of either gene is markedly reduced upon mutation of the other [6]. However, mutation of *TP53* appeared to exhibit slightly increased selection after mutation of *CTNNB1.* Such non-reciprocality likely arises as a consequence of gene-regulatory state change that renders the effect of the combination of mutations different due to specific regulatory adaptation to either individual mutation. Such asymmetry is supported by the observation of relatively rare but nevertheless present co-mutation events in several other cohorts, including those from Asia [23,24]. These candidate epistatic relationships were not significant in TCGA-LIHC. Significance was not retained after Benjamini-Hochberg adjustment (**S2 Fig**), likely reflecting limited statistical power from sparse co-mutation counts and the large number of pairwise tests. Therefore, current epistasis results are best viewed as hypothesis generating rather than confirmatory (**S1 Fig**).

Survival analysis in TCGA-LIHC complemented the CES analysis by showing that *TP53* mutation status was associated with worse overall survival in this independent cohort. However, this association did not remain significant after multiple-testing adjustment across the selected genes, and *TP53* was not significantly associated with disease-free survival in the Cox model. Other selected genes, including *CTNNB1*, *JAK1*, *STAT3*, and *IL6ST*, did not show significant survival associations after Cox modeling and multiple-testing adjustment. For *P4HA1*, survival modeling was not reliable because only one mutation-positive TCGA-LIHC patient had usable survival annotation. Because survival data were not available to us for CLCA, these clinical associations should be interpreted as cohort-specific rather than as direct validation of CLCA-derived effect-size estimates.

Our study has several limitations. The relatively small number of cases carrying individual somatic mutations reduces statistical power, particularly for survival analyses. WGS and WXS datasets are not directly equivalent, especially for non-coding regions and low prevalence variants. Moreover, cancer effect size and survival analyses were conducted in separate cohorts, introducing potential confounding from population-level socioeconomic heterogeneity other than race and ethnicity. Finally, biological complexity may obscure the relationship between mutation impact and patient prognosis, as high-effect mutations represent increased survival and proliferation of the cancer cell lineage, traits that may anticorrelate with patient survival but that do not necessarily translate into worse outcomes; other phenotypes, such as invasiveness or metastatic potential, may play much larger roles in determining patient survival.

Despite these limitations, our findings can help prioritize biologically plausible candidate drivers for future functional and clinical investigation. The association between *TP53* mutations and poor survival reinforces their established prognostic value and underscores the utility of genomic profiling in HCC. In addition, the prioritization of *P4HA1*, *STAT3*, *IL6ST*, and ENSG00000291313 highlights pathways and loci that warrant mechanistic follow-up, particularly to determine whether the specific variants identified here alter protein function, regulatory activity, or tumor-cell fitness.

## Conclusion

In summary, our analysis highlights population-specific vulnerabilities in Chinese HCC and reveals both common and rare high-effect mutations with potential cooperative interactions across cohorts. Canonical HCC drivers, including *TP53*, *CTNNB1*, and *TERT* promoter mutations, showed strong inferred positive selection, supporting the validity of the cancer-effect framework. In addition, lower-prevalence events in *P4HA1*, *STAT3*, *IL6ST*, and ENSG00000291313 were prioritized as candidate high-effect mutations or loci. Together, these findings extend the insights of the Chinese Liver Cancer Atlas by prioritizing candidate drivers by inferred effect size and providing a framework for future functional validation, survival analysis in larger cohorts, and informed evaluation of clinical relevance.

## Supporting information

S1 Fig Independently estimated and epistatic effects of gene mutations within the HCC-CLCA cohort. Independently estimated cancer effects (filled circles) and nominal (likelihood-ratio test *P* values < 0.05) gene-specific pairwise synergistic (red) or antagonistic (blue) epistatic effects (arrowheads) for amino-acid-changing mutations in the HCC-CLCA cohort for (**A**) *PIK3CA* and *CDKN2A*, (**B**) *CTNNB1* and *JAK1*, (**C**) *CTNNB1* and *TP53*, (**D**) ENSG00000291313 and *P4HA1*, and (**E**) *JAK1* and *TP53*.
(TIF)

S2 Fig Independently estimated and epistatic effects of gene mutations within the LIHC-TCGA cohort. Independently estimated cancer effects (filled circles) and nominal (likelihood-ratio test *P* values < 0.05) gene-specific pairwise synergistic (red) or antagonistic (blue) epistatic effects (arrowheads) for amino-acid-changing mutations in the LIHC-TCGA cohort for (**A**) *F5* and *TTN*, and (**B**) *F5* and *TP53*.
(TIF)

S3 Fig Survival outcomes between tumors with and without nonsynonymous mutations in selected driver genes within the HCC-CLCA cohort. Kaplan-Meier curves were plotted for (**A**) *TP53* overall survival (OS), (**B**) *TP53* disease-free survival (DFS), (**C**) *CTNNB1* OS, (**D**) *CTNNB1* DFS, (**E**) *JAK1* OS, (**F**) *JAK1* DFS, (**G**) *IL6ST* OS, (**H**) *IL6ST* DFS, (**I**) *STAT3* OS, and (**J**) *STAT3* DFS. Mutation-positive tumors are shown in cyan, and wild-type tumors are shown in dark gray. Shaded regions indicate 95% confidence intervals. Vertical tick marks indicate censored observations. Log-rank *P* values shown within each plot represent nominal univariate comparisons between mutation-positive and wild-type groups. The risk table below each plot shows the number of patients at risk at each time point; values in parentheses indicate the cumulative number of censored patients up to that time point. Time is shown in months from diagnosis.
(TIF)

S4 Dataset Variant-level cancer effect size results for the CLCA and TCGA-LIHC cohorts. Gene annotations were filled using the corresponding Hugo_Symbol values from the original preloaded MAF files matched by variant_id. A cohort column was added to indicate whether each result came from the CLCA-HCC or TCGA-LIHC analysis.
(CSV)

S5 Dataset Gene-level epistasis analysis results for the CLCA and TCGA-LIHC cohorts. Genes with at least 3 total variants were included in the epistasis analysis if they had at least three total included amino-acid change variants in the corresponding cohort-specific variant-level CES result table.
(CSV)
